# Knowledge, Attitudes, and Practices of Adult Vaccination Among Medical Students in Western India

**DOI:** 10.7759/cureus.108777

**Published:** 2026-05-13

**Authors:** Ankita Sharma, Yachana Choudhary, Parshika Panwar, Nitasha Sharma, Sudarshana Sudarshana, Sweta Sinku, Nisha Kumari, Dewesh Kumar

**Affiliations:** 1 Department of Microbiology, Government Medical College, Pali, IND; 2 Department of Community Medicine, Government Medical College, Pali, IND; 3 Department of General Medicine, Government Medical College, Pali, IND; 4 Department of Community Medicine, Rajendra Institute of Medical Sciences, Ranchi, IND

**Keywords:** adult vaccination, awareness, immunization, medical students, vaccine hesitancy

## Abstract

Background and aim: Immunization is one of the most effective public health interventions, significantly reducing morbidity and mortality from infectious diseases worldwide. While childhood vaccination programs are well-established, adult immunization remains underutilized, particularly in developing countries like India. This study aimed to assess the knowledge, attitudes, and practices (KAP), along with beliefs, misconceptions, and barriers related to adult vaccination among medical students in Western Rajasthan, specifically at Government Medical College, Pali, and to examine their association with selected socio-demographic factors.

Methods: A cross-sectional study was conducted among 575 undergraduate medical students at a tertiary care center using a pre-tested, semi-structured questionnaire. Participants were recruited through convenience sampling, and data were analyzed using descriptive and inferential statistics.

Results: Overall, 69.6% of participants were aware of the adult vaccination schedule. Knowledge was higher for commonly administered vaccines, such as COVID-19 (96.5%) and hepatitis B (92.3%), but lower for newer vaccines, such as monkeypox (56.5%) and respiratory syncytial virus (55.1%). Attitudes were largely favorable, with 98.6% recognizing the importance of adult vaccination and 94.4% supporting its inclusion in national programs. However, misconceptions were widespread, with 72.7% believing vaccines may cause serious complications. Although all participants reported receiving at least one vaccine after 18 years (primarily COVID-19), only 61.6% were willing to receive adult vaccines, indicating a gap between knowledge and practice. Lack of awareness and fear of side effects were the most commonly reported barriers.

Conclusion: Despite moderate knowledge and positive attitudes, significant misconceptions and gaps in uptake persist among medical students. Targeted educational interventions and strengthened curriculum integration are needed to improve vaccine literacy and acceptance. Findings are limited by a single-center design, convenience sampling, and self-reported data.

## Introduction

Immunization stands as one of the greatest achievements in global health, preventing millions of deaths annually. According to the World Health Organization (WHO), vaccines safeguard an estimated 3.5 to 5 million people each year by shielding individuals from deadly diseases like diphtheria, tetanus, pertussis, influenza, and measles [1]. Riedel described Edward Jenner’s pioneering contribution to the development of immunization [2]. Vaccination is not only a fundamental component of primary health care but also an undeniable human right and a highly cost-effective health investment. Vaccines are essential for preventing the spread of infectious diseases, which protects the community as a whole in addition to protecting individuals from potentially fatal illnesses [3]. As a result, international organizations, particularly the WHO, have made significant strides in promoting immunization worldwide [4].

In India, immunization efforts began with the WHO Expanded Program on Immunization (EPI) in 1978, followed by the launch of the Universal Immunization Program (UIP) in 1985 [5]. These initiatives are primarily aimed at decreasing morbidity and mortality from vaccine-preventable diseases and have contributed to substantial improvements in healthcare delivery systems, including vaccine production, distribution, and cold chain maintenance [6]. However, these programs have primarily focused on children, adolescents, and pregnant women, while adult immunization remains relatively neglected.

As individuals age, immunity declines, which increases susceptibility to various infections, particularly among those with comorbidities or weakened immune systems [7]. Globally, adult vaccination is gaining recognition; however, it is not yet fully integrated into routine healthcare in many countries, including India [3,8]. Unlike pediatric immunization, which is guided by well-established national policies, adult immunization lacks a comprehensive and unified framework. Low vaccination coverage among adults can be attributed to multiple factors, including limited awareness among healthcare providers and the general population, concerns regarding vaccine safety and efficacy, and the absence of standardized national guidelines [9-11]. In addition, beliefs and misconceptions about vaccine safety, as well as perceived barriers such as fear of side effects, lack of access, and inadequate recommendations from healthcare providers, further contribute to low uptake of adult vaccines [12,13].

Although recommendations from organizations such as the Centers for Disease Control and Prevention, WHO, and the Association of Physicians of India provide some guidance, India still lacks a structured national policy for adult immunization [7]. Addressing this gap is essential for improving public health outcomes. Primary healthcare providers and family physicians play an influential role in promoting adult immunization. They are often the first point of contact for patients and are well-positioned to assess vaccination status, address vaccine hesitancy, and provide appropriate guidance [14]. Adult vaccination is particularly important for preventing infections such as influenza, pneumonia, and shingles, especially among high-risk groups [15]. Furthermore, vaccination is essential for individuals in certain occupations or those traveling internationally, as it helps prevent disease transmission and reduces healthcare burden [16,17].

In this context, medical students represent a key group, as they are future healthcare providers who will influence vaccination practices in the community. Their knowledge, attitudes, and practices regarding adult immunization are critical for improving vaccine uptake. Understanding their beliefs, misconceptions, and perceived barriers toward adult vaccination is equally important for designing targeted educational interventions. However, limited evidence exists regarding these aspects among medical students in India, highlighting a critical research gap.

## Materials and methods

Study design, setting, and participants

A cross-sectional study was carried out at Government Medical College, Pali, Rajasthan, for a period of eight months (January 2025-August 2025). The study included undergraduate medical students (MBBS) from all academic years (first to fourth year) as well as interns enrolled at the institution.

Inclusion and exclusion criteria

All medical students who were present during the study period and consented to participate were included in this study. Students who were not willing to participate in the study were excluded.

Sample size calculation

The sample size was calculated using the standard formula: n = z²pq/d²​​​​​​​. Here z = 1.96 at a 95% confidence level; p is prevalence, which is 52% (based on findings from previous studies); q = 1-p; and d = 5% allowable error. The calculated sample size was 383. Considering a 20% non-response rate due to the online nature of data collection, the minimum required sample size estimated was 460. Although the minimum calculated sample size was 460, all eligible students available during the study period were invited to participate, resulting in a final sample of 575 participants. A convenience sampling method was used, wherein the questionnaire was circulated online, and participants who responded during the study period were included (table in appendix).

Data collection tools

Data were collected using a semi-structured questionnaire administered via Google Forms (Mountain View, CA: Google LLC). The questionnaire was developed based on previously published literature and guidelines on adult immunization. It was reviewed by subject experts for content validity, internal consistency of the questionnaire was assessed using Cronbach’s alpha. It covered socio-demographic characteristics and assessed knowledge, awareness, attitudes, and practices related to adult vaccination. It also included questions about vaccine-preventable diseases, recommended adult vaccination schedules, beliefs and misconceptions, perceived barriers, and self-reported vaccination status.

Data collection procedure

The questionnaire link was circulated to participants via online platforms by the principal investigator (PI). Prior to participation, informed consent was obtained electronically. The consent form was provided in both English and the local language to ensure better comprehension.

Data analysis

Data were entered and analyzed using Epi Info software version 7.2 (Atlanta, GA: Centers for Disease Control and Prevention) and Microsoft Excel version 16 (Redmond, WA: Microsoft Corp.). Descriptive statistics were used to summarize the data, and results were expressed as frequencies and percentages. Knowledge scores were calculated based on correct responses and categorized as good (≥75% correct responses), moderate (50-74%), and poor (<50%). Inferential analysis was performed using the chi-square test to assess associations between socio-demographic variables and awareness levels. A p<0.05 was considered statistically significant.

Quality assurance

To ensure data quality, the study protocol underwent peer review prior to implementation. The questionnaire was pilot-tested among a small group of residents to assess clarity and relevance. Additionally, 10% of the collected responses were randomly cross-checked by the PI to verify consistency and accuracy.

Ethical considerations

The study was approved by the Institutional Ethics Committee of Government Medical College, Pali, with approval no. GMC/IEC/2025/192. Participation was voluntary, and the confidentiality of the respondents was maintained. Informed consent was obtained from all participants, and they were assured of their right to withdraw from the study at any stage without any consequences.

## Results

The questionnaire demonstrated good internal consistency, with a Cronbach’s alpha value of >0.70. A total of 575 medical students participated in the study. The study population included 289 (50.3%) females and 286 (49.7%) males. The majority of participants belonged to the 22-25 years age group (60.3%). Most students were Hindu (95.3%), followed by Muslim (2.8%) and Jain (1.9%). Nearly all participants were unmarried (98.8%). In terms of residence, 60.7% were from urban areas and 39.3% from rural areas. The socio-demographic characteristics of the participants are in Table 1.

**Table 1:** Socio-demographic characteristics of study participants (n=575). n: frequency

Variables	Category	n (%)
Age (years)	18-21	217 (37.7)
	22-25	347 (60.3)
	26-29	11 (1.9)
Gender	Male	286 (49.7)
	Female	289 (50.3)
Religion	Hindu	548 (95.3)
	Jain	11 (1.9)
	Muslim	16 (2.8)
Residential	Rural	226 (39.3)
	Urban	349 (60.7)

Awareness of adult vaccination

**Figure 1:**
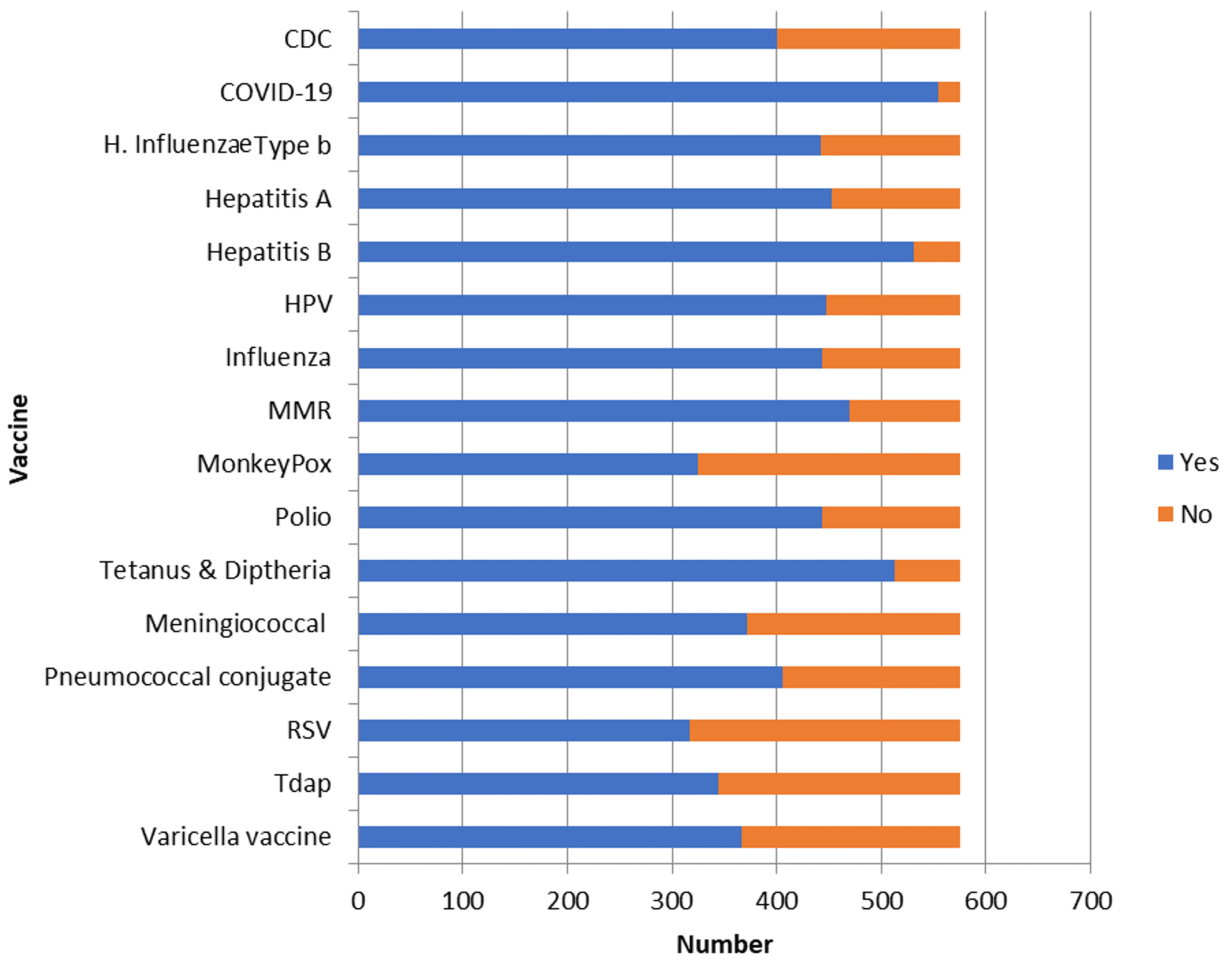
Showing the awareness regarding adult immunization. CDC: Centers for Disease Control and Prevention; HPV: human papillomavirus; MMR: measles, mumps, rubella; RSV: respiratory syncytial virus; Tdap: tetanus, diphtheria, and acellular pertussis

Overall, 400 (69.6%) participants were aware of the recommended adult vaccination schedule. Awareness was high for commonly known vaccines, including COVID-19 (96.5%), hepatitis B (92.3%), and tetanus and diphtheria (89.2%). Moderate awareness was observed for vaccines such as hepatitis A (78.8%), human papillomavirus (77.7%), influenza (77%), and *Haemophilus influenzae* type b (76.9%). However, awareness was comparatively lower for newer vaccines such as monkeypox (56.5%) and respiratory syncytial virus (55.1%) (Figure 1).

Beliefs and misconceptions

A substantial proportion of participants (72.7%) believed that vaccines may cause serious complications, indicating the presence of misconceptions and negative beliefs regarding vaccine safety. Additionally, 7.8% believed that vaccines could lead to sterility. Only 15.8% reported not believing in any myths. Minor concerns such as common side effects (fever, rash) were reported by 2.8%, while 0.9% reported other rare beliefs (Table 2).

**Table 2:** Beliefs and myths regarding adult vaccination (n=575). n: frequency

Belief/myth	n (%)
Vaccines may cause serious complications	418 (72.7)
Impotency/sterility due to vaccines	45 (7.8)
Do not believe in any myth	91 (15.8)
Minor side effects (fever, rash, etc.)	16 (2.8)
Other rare beliefs	5 (0.9)

Barriers to adult vaccination

The most commonly reported barrier to adult vaccination was lack of awareness (51.1%), followed by fear of side effects (23.7%). Other barriers included lack of time (14.6%), cost concerns (5.6%), and belief in myths (5.0%) (Table 3).

**Table 3:** Barriers to adult vaccination (practice gap analysis) (n=575). n: frequency

Barrier	n (%)
Not aware about vaccines	294 (51.1)
Fear of side effects	136 (23.7)
No time	84 (14.6)
Cost concerns	32 (5.6)
Belief in myths	29 (5.0)

Knowledge, attitudes, and practices

Overall, 69.6% of participants were aware of the adult vaccination schedule, while 30.4% were not aware. Based on composite scoring, 46.6% had good knowledge, 34.3% had moderate knowledge, and 19.1% had poor knowledge. High awareness of commonly administered vaccines was observed in 61.9%, whereas awareness of newer vaccines was lower (54.3%) (Table 4).

**Table 4:** Knowledge, attitude, practice analysis of adult immunization among undergraduate students (n=575). n: frequency; RSV: respiratory syncytial virus

Domain	Indicator	Category	n (%)
Knowledge	Awareness of adult vaccination schedule	Yes	400 (69.6)
		No	175 (30.4)
	Knowledge level (composite)	Good	268 (46.6)
		Moderate	197 (34.3)
		Poor	110 (19.1)
	Awareness of common vaccines (≥75%)	High awareness group	356 (61.9)
	Awareness of newer vaccines (RSV, monkeypox, Tdap)	Low awareness group	312 (54.3)
Attitude	Adult vaccination important for healthcare professionals	Yes	567 (98.6)
		No	8 (1.4)
	Should be part of National Immunisation Programme	Yes	543 (94.4)
		No	32 (5.6)
	Would recommend to family members	Yes	495 (86.1)
		No	80 (13.9)
	Presence of myths/beliefs	Yes (any myth present)	484 (84.2)
		No myth	91 (15.8)
Practice	Received any vaccine after 18 years	Yes	575 (100)
	Experience of side effects	Yes	140 (24.3)
		No	435 (75.7)
	Willingness to take adult vaccines	Yes	354 (61.6)
		Maybe	175 (30.4)
		No	46 (8.0)
	Ever searched for adult vaccines	Yes	391 (68.0)
		No	184 (32.0)

With respect to attitude, a large majority (98.6%) agreed that adult vaccination is important for healthcare professionals, and 94.4% supported its inclusion in the National Immunisation Programme. Additionally, 86.1% reported that they would recommend adult vaccination to their family members. However, 84.2% reported the presence of at least one myth or belief, while only 15.8% reported no such misconceptions (Table 4).

Regarding practices, all participants (100%) reported receiving at least one vaccine after the age of 18 years, largely due to widespread COVID-19 vaccination during the national immunization campaign. A total of 24.3% reported experiencing side effects following vaccination. In terms of willingness, 61.6% were willing to receive adult vaccines, 30.4% were uncertain, and 8.0% were unwilling. Furthermore, 68.0% reported that they had searched for information regarding adult vaccines (Table 4).

Association with socio-demographic variables

A statistically significant association was observed between age and awareness of adult vaccination (p<0.001), with higher awareness among participants aged 22-25 years. Religion was also found to be marginally significantly associated with awareness (p=0.046); however, this finding should be interpreted with caution due to unequal distribution among groups. No statistically significant association was observed with gender (p=0.862), marital status (p=0.078), or place of residence (p=0.483) (Table 5).

**Table 5:** Association between socio-demographic characteristics and awareness about the recommended adult vaccination schedule. *The statistical test used is the chi-square test. #P<0.05 is the significance level. n: frequency

Variables	Category	Yes, n (%)	No, n (%)	χ²	p-Value^#^
Age (years)*	18-21	123 (56.7)	94 (43.3)	28.3	<0.001
	22-25	267 (76.9)	80 (23.1)		
	26-29	10 (90.9)	1 (9.1)		
Gender*	Female	202 (69.9)	87 (30.1)	0.03	0.862
	Male	198 (69.2)	88 (30.8)		
Marital status*	Married	7 (100)	0 (0)	3.10	0.078
	Single	393 (69.2)	175 (30.8)		
Religion*	Hindu	380 (69.3)	168 (30.7)	6.17	0.046
	Jain	11 (100)	0 (0)		
	Muslim	9 (56.3)	7 (43.8)		
Residential*	Rural	161 (71.2)	65 (28.8)	0.49	0.483
	Urban	239 (68.5)	110 (31.5)		

## Discussion

This study assessed critical knowledge, attitudes, and practices of adult vaccination among 575 medical students, with a gender distribution of 50.3% female and 49.7% male. The present study demonstrates a clear misalignment across these domains among medical students. Although 69.6% of participants were aware of adult immunization, only 46.6% demonstrated good knowledge, indicating that awareness does not necessarily translate into comprehensive understanding. This gap suggests that knowledge acquisition is partial and likely influenced by selective exposure rather than structured learning.

The present study demonstrated moderate knowledge of adult immunization, with 69.6% aware and only 46.6% demonstrating good knowledge. Similar patterns have been reported in earlier studies, including one in which 97.2% of medical students reported awareness of the human papillomavirus (HPV) vaccine, despite lower uptake, reflecting a gap between knowledge and practice [18,19]. Awareness varied across vaccines, with higher awareness observed for commonly administered vaccines compared to newer ones, consistent with previous studies indicating greater familiarity with vaccines included in national programs [8,20,21]. In contrast, awareness of newer vaccines such as monkeypox and respiratory syncytial virus (RSV) was relatively low, which aligns with literature suggesting that newly introduced or less-publicized vaccines are less well-known among both healthcare professionals and the general population [22]. A review on adult vaccination in India similarly highlights that awareness remains limited overall, particularly for vaccines not included in national programs [23]. Additionally, prior research among healthcare professionals has shown partial awareness of adult immunization concepts [14]. Overall, these findings align with existing literature reporting inadequate and variable knowledge regarding adult vaccination among healthcare trainees and professionals [18,24,25].

Attitudes toward adult vaccination were largely positive, with 98.6% recognizing its significance and 94.4% supporting its inclusion in national programs, indicating strong acceptance at a conceptual level. However, the coexistence of such favorable attitudes with a high prevalence of misconceptions (84.2%) suggests that these attitudes may be superficial or normative rather than evidence-based. In other words, while students conceptually support vaccination, their limited confidence in vaccine safety may constrain informed decision-making. Similarly, in a study by Sondankar et al., participants demonstrated favorable attitudes toward vaccination [19]. Studies among healthcare trainees further show that willingness to vaccinate remains moderate despite adequate awareness, reflecting persistent hesitancy driven by misconceptions and lack of confidence [26,27]. This disconnect implies that positive attitudes alone are insufficient to ensure appropriate vaccination practices unless supported by accurate knowledge and trust in vaccine safety.

In terms of practices, although 100% of participants reported receiving vaccination after 18 years, this was largely attributable to COVID-19 vaccination, reflecting the influence of external mandates and public health urgency rather than routine health-seeking behavior. In contrast, only 61.6% expressed willingness to receive adult vaccines, with a considerable proportion remaining uncertain. This discrepancy highlights a clear attitude-practice gap, where positive perceptions do not consistently translate into proactive vaccination behavior. The findings suggest that misconceptions, limited knowledge, and perceived barriers continue to influence decision-making, thereby restricting voluntary uptake.

Misconceptions were highly prevalent in the present study, with a substantial proportion of participants believing that vaccines could cause serious complications. Fear of side effects and inadequate awareness emerged as key barriers, likely contributing to the lower willingness to receive adult vaccines despite relatively high awareness levels. Consistent with this, the most common barriers identified were lack of awareness and fear of side effects, followed by time constraints and cost concerns, aligning with findings from studies conducted in India and globally [19,21,28]. These concerns are further supported by a study conducted in Delhi, which reported fear of side effects (51.41%) and lack of awareness (49.46%) as the leading causes of vaccine hesitancy [26]. These findings suggest that both cognitive factors (misconceptions, risk perception) and practical constraints (time, cost) could play a role in shaping vaccination behavior.

A statistically significant association was observed between age and awareness, with higher awareness among older students, likely due to greater academic and clinical exposure, as reported in a previous study [29]. No significant association was found with gender or residence, suggesting that awareness is more strongly influenced by educational exposure rather than demographic characteristics [29,30].

These findings have important implications for clinical practice, policy, and future research. The presence of misconceptions despite positive attitudes suggests that medical students may not be adequately prepared to counsel patients on vaccine safety, which could affect vaccine advocacy in clinical settings. Integrating structured training on adult immunization and vaccine communication into the medical curriculum may help bridge this gap. At the policy level, the findings support the need for clearer national guidelines and inclusion of adult vaccination in routine healthcare strategies. For future research, multi-center studies and interventional designs are needed to evaluate the effectiveness of educational programs in improving knowledge, correcting misconceptions, and enhancing vaccine uptake.

Limitation

This study has several limitations. The use of a self-reported questionnaire may introduce recall and social desirability bias. The inclusion of students from different phases of the MBBS curriculum (from first year to internship) may have led to variability in knowledge and awareness levels due to differences in academic and clinical exposure. Additionally, the use of the convenience sampling method and online data collection may result in selection bias. Being a single-center study, the findings may not be generalizable to other settings. Additionally, the lack of detailed validation of the questionnaire and the absence of multivariable analysis may limit the robustness of the findings.

## Conclusions

This study demonstrates that medical students have moderate knowledge and generally favorable attitudes toward adult vaccination; however, significant misconceptions and gaps in practice persist. The coexistence of favorable perceptions with incorrect beliefs about vaccine safety highlights a disconnect between awareness and accurate understanding. These findings underscore the need for targeted educational interventions and stronger integration of adult immunization into the medical curriculum. Addressing these gaps is essential to improve vaccine literacy, enhance acceptance, and prepare future healthcare providers for effective vaccine advocacy.

## Appendices

**Table 6:** Questionnaire assessing awareness of adult vaccination GMC: Government Medical College; MMR: measles, mumps, rubella; RSV: respiratory syncytial virus; Tdap: tetanus, diphtheria, and acellular pertussis; IPV: inactivated polio vaccine; Hib: *Haemophilus influenzae *type b

Question	Response (tick/write)																						
1. Age (years)																							
2. Gender	Male						Female										Others						
3. Marital status	Single										Married												
4. Batch	2020		2021					2022						2023								2024	
5. Roll number																							
6. Religion	Hindu		Muslim					Christian						Jain								Other	
7. Residential	Rural												Urban										
8. Aware of the CDC vaccination schedule?	Yes												No										
9. Aware of the COVID-19 vaccine for adults?	Yes												No										
10. Aware of the Hib vaccine for adults?	Yes												No										
11. Aware of the hepatitis A vaccine?	Yes												No										
12. Aware of the hepatitis B vaccine?	Yes												No										
13. Aware of the HPV vaccine?	Yes												No										
14. Aware of the influenza vaccine?	Yes												No										
15. Aware of the MMR vaccine?	Yes												No										
16. Aware of the monkeypox vaccine?	Yes												No										
17. Aware of the IPV vaccine?	Yes												No										
18. Aware of the tetanus and diphtheria vaccine?	Yes												No										
19. Aware of the meningococcal vaccine?	Yes												No										
20. Aware of the pneumococcal vaccine?	Yes												No										
21. Aware of the RSV vaccine?	Yes												No										
22. Aware of the Tdap vaccine?	Yes												No										
23. Aware of the varicella vaccine?	Yes												No										
24. Ever immunized? Mention vaccine																							
25. Side effects after vaccination?	Yes												No										
26. Willing to take vaccines?	Yes						No										Maybe						
27. Is adult vaccination important?	Yes												No										
28. Source of information	Print			Social media									Doctors							Med school			
29. Ever Googled about vaccines?	Yes												No										
30. Reason for not being vaccinated	Myth	Not aware							No time						Cost						Fear		
31. Myth believed	Impotency					Serious complications												Other					
32. Should be in the national program?	Yes												No										
33. Any comorbidity?	Yes												No										
34. Type of comorbidity	Diabetes				HTN					Allergy						Heart disease							Other
35. Substance dependence	Smoking				Alcohol					Tobacco						Weed							None
36. Comorbid patients need vaccines?	Yes											No											
37. Community protection?	Yes						No												Other				
38. Recommend to family?	Yes											No											
39. Aware of vaccines in GMC Pali?																							
